# A Hybrid Stacked Restricted Boltzmann Machine with Sobel Directional Patterns for Melanoma Prediction in Colored Skin Images

**DOI:** 10.3390/diagnostics13061104

**Published:** 2023-03-14

**Authors:** A. Sherly Alphonse, J. V. Bibal Benifa, Abdullah Y. Muaad, Channabasava Chola, Md Belal Bin Heyat, Belal Abdullah Hezam Murshed, Nagwan Abdel Samee, Maali Alabdulhafith, Mugahed A. Al-antari

**Affiliations:** 1 School of Computer Science and Engineering, Vellore Institute of Technology, Chennai 600127, India; 2 Department of Studies in Computer Science and Engineering, Indian Institute of Information Technology, Kottayam 686635, India; 3 Department of Studies in Computer Science, University of Mysore, Manasagangothri, Mysore 570006, India; 4 IoT Research Center, College of Computer Science and Software Engineering, Shenzhen University, Shenzhen 518060, China; 5 Department of Information Technology, College of Computer and Information Sciences, Princess Nourah bint Abdulrahman University, P.O. Box 84428, Riyadh 11671, Saudi Arabia; 6 Department of Artificial Intelligence, College of Software and Convergence Technology, Daeyang AI Center, Sejong University, Seoul 05006, Republic of Korea

**Keywords:** skin melanoma, AI-based detection, Restricted Boltzmann Machines, Sobel image processing

## Abstract

Melanoma, a kind of skin cancer that is very risky, is distinguished by uncontrolled cell multiplication. Melanoma detection is of the utmost significance in clinical practice because of the atypical border structure and the numerous types of tissue it can involve. The identification of melanoma is still a challenging process for color images, despite the fact that numerous approaches have been proposed in the research that has been done. In this research, we present a comprehensive system for the efficient and precise classification of skin lesions. The framework includes preprocessing, segmentation, feature extraction, and classification modules. Preprocessing with DullRazor eliminates skin-imaging hair artifacts. Next, Fully Connected Neural Network (FCNN) semantic segmentation extracts precise and obvious Regions of Interest (ROIs). We then extract relevant skin image features from ROIs using an enhanced Sobel Directional Pattern (SDP). For skin image analysis, Sobel Directional Pattern outperforms ABCD. Finally, a stacked Restricted Boltzmann Machine (RBM) classifies skin ROIs. Stacked RBMs accurately classify skin melanoma. The experiments have been conducted on five datasets: Pedro Hispano Hospital (PH2), International Skin Imaging Collaboration (ISIC 2016), ISIC 2017, Dermnet, and DermIS, and achieved an accuracy of 99.8%, 96.5%, 95.5%, 87.9%, and 97.6%, respectively. The results show that a stack of Restricted Boltzmann Machines is superior for categorizing skin cancer types using the proposed innovative SDP.

## 1. Introduction

An extremely dangerous type of melanoma, termed malignant melanoma, develops in skin cells known as melanocytes [1]. Melanocytes are skin cells that are located in the top layer of skin. They are responsible for the production of the pigment known as melanin, which is responsible for the color of skin. Eumelanin and pheomelanin are the two forms of melanin that can be found in the human body. The melanocytes are stimulated to create more melanin when skin is damaged by ultraviolet (UV) radiation from the sun or tanning beds. However, only the eumelanin pigment in the skin makes an effort to protect the skin by causing the skin to darken or tan. Melanoma arises when DNA is damaged from sunburns or tanning beds as a result of ultraviolet radiation, which then causes alterations (mutations) in the melanocytes, which ultimately leads to uncontrolled cellular proliferation [2]. This form of skin cancer is riskier than other common types including basal cell carcinoma and squamous cell carcinoma. If it is not treated at an early stage, it has the potential to swiftly spread to other organs, making it more difficult to cure [3]. 

In the United States in 2022, it was anticipated that there would be 99,780 recently diagnosed occurrences of invasive melanoma and 97,920 newly diagnosed instances of in situ melanoma and that cancer would claim the lives of 7650 individuals [4]. Occurrence rates are greater in women than in men prior to the age of 50, whereas, after that age, they are increasingly higher in men. This is mainly due to age gaps in historical workplaces and outdoor exposure to UV radiation, as well as the usage of in-door tanning among many young women. Men have a higher risk of developing skin cancer after the age of 50. Variations in the methods of early detection and the utilization of health care could also be contributing factors. About 300,000 new cases of melanoma were reported worldwide in 2018, making it the most frequent malignancy in both men and women [2]. Over a million new cases of basal cell carcinoma (BCC) and squamous cell carcinoma (SCC) were diagnosed in 2018, making them the second and third most common forms of skin cancer after melanoma [5]. A greater number of cases of skin cancer are diagnosed annually compared to any other type of cancer in the United States. The good news is that early detection considerably increases the likelihood of a successful treatment. Without spreading, a 99% 5-year survival rate is reported for melanoma patients [5]. When it spreads to other parts of the body, the prognosis is not as good. However, the success of a dermatologist’s diagnosis depends heavily on their experience and training, as the earliest signs of skin cancer are usually not obvious. Skin cancer of the non-melanoma variety is quite prevalent. A total of 300,000 new cases of melanoma, which is ranked 19th among the most prevalent cancer kinds, were discovered in 2018. In 2019 alone, there were approximately 2490 females and 4740 males who lost their lives to melanoma. There are an estimated 2–3 million new cases annually [4], with over 40% attributable to disorders other than melanoma. It is especially common in those with very fair skin. A lower mortality rate can be achieved with earlier cancer detection. Better treatment for the patient is another benefit.

Clinicians typically do screenings for skin cancer via eye inspection, which is not only more time-consuming but also more prone to error and subjectivity. Dermoscopy is a noninvasive imaging technology that removes the skin’s surface reflection, allowing for better illumination and magnification of skin abnormalities. Melanoma is generally predicted by using the ABCDE rule. Specialists assess a mole based on its Asymmetry, Border, Dimension, Color, and Edge. Yet, diagnoses based solely on visual inspection tend to be more off-base. Different methods have been proposed in the literature. However, diagnosing the specific form of skin cancer might be challenging. Reduced mortality rates from skin cancer can be achieved with diligent early detection efforts. Professionals need some time to make a correct early diagnosis. However, the dermatologist accuracy in detecting melanoma using dermoscopy images was lower than 80% in traditional clinical settings [6,7,8]. Using machine learning techniques [9,10,11], this analysis could be automated, leading to a framework in medicine that would provide experiential relevance, improve clinical accuracy, aid physicians in interacting objectively, reduce errors caused by human stress, and lower mortality rates [12,13,14,15]. One step in the right direction is the development of a machine-learning algorithm that can distinguish between malignant and benign lesions [16,17]. To identify cancerous skin lesions as soon as possible, this work makes use of Machine Learning, and deep learning algorithms to reliably categorize pigmented lesions in dermoscopic skin images.

Skin malignancies develop as a result of aberrant cell growth and can spread to other body parts [1]. The PH2 dataset divides skin cancer into three major types. They are atypical nevus, melanoma, and common nevus. A common nevus is a benign skin growth that occurs because melanocytes, or pigment cells, proliferate. It appears as a little dot in the epidermis, and determining whether it is benign or malignant is exceedingly challenging. A typical application of biopsy is also not acceptable. The creation of a completely automated melanoma identification system is crucial for assisting dermatologists with diagnosis [2]. 

In general, the classification of skin cancer is difficult due to the presence of artifacts, differences in picture resolution, and fewer distinguishing characteristics across the many forms of cancer. As a result of these issues, the efficient framework may be deemed an ideal model for skin cancer classification due to its compound scaling property. This will help to strengthen the accuracy of the classification. Within the scope of this study, an end-to-end framework for the effective and accurate classification of skin lesions is provided. The proposed framework is composed of preprocessing, segmentation, feature extraction, and classification modules. During the preprocessing stage, the DullRazor algorithm is utilized to remove hairs from the material. This assists in reducing the influence of artifacts caused by the presence of hairs in skin imaging. After that, a semantic segmentation strategy that makes use of Fully Connected Neural Networks (FCNNs) is developed to extract precise and clear Regions of Interest (ROIs). After that, the key features included within the ROIs are retrieved by utilizing an enhanced version of the Sobel Directional Pattern (SDP), a methodology we are proposing here in this research to extract relevant features contained within skin images. When it comes to analyzing skin images, the Sobel Directional Pattern approach is preferable to the more conventional feature extraction strategy known as the ABCD rule [18]. As a last step, a stacked Restricted Boltzmann Machine, also known as a stacked RBM, is introduced as a solution for the classification of skin ROIs. The stacked RBMs are being presented due to their outstanding performance in the classification of skin melanoma [19]. 

Artificial intelligence (AI) and associated technologies are starting to be adopted by healthcare organizations as they become increasingly widespread in the industrial and medical sectors [20,21,22,23,24]. Studies [25,26,27,28,29,30,31,32,33] have proven that AI is as good as, or better than, human doctors when it comes to medical diagnosis. Recently, machine learning and deep learning algorithms [18] have been more accurate than radiologists in detecting malignant tumors. They are also aiding researchers in figuring out how to assemble study populations for costly clinical trials. Since modern graphics processing units (GPUs) are capable of handling massive amounts of data, such models may also have hundreds of thousands of hidden features. One of the most common uses of deep learning in healthcare is the detection of potentially malignant tumors in medical images. The field of radiomics, which uses deep learning to reveal hidden clinically relevant patterns in imaging data, is gaining traction. Typically, radiomics and deep learning are used together in oncology-specific picture analysis. With both, CAD systems can make more accurate and precise diagnoses. Artificial intelligence has been supported by many researchers recently as a means to automatically detect and diagnose skin disorders [34,35,36,37,38]. Gonzalez-Castro et al. [39] suggested categorizing skin lesions using color and texture descriptors based on morphology. In their method, the hue and mathematical morphology of the color texture are examined. Additionally, they have employed Kohonen Self-Organizing Maps (SOM). They do not segregate anything at all. For each pixel, mathematical morphology generates a descriptor. Clusters are produced in SOM. The descriptors do not depend on location. Color Adaptive Neighborhoods are represented mathematically through morphology. However, it is never easy to get a perfect mapping using this method. Using the ABCD rule, Kasmi et al. [3] used ABCD instruction of dermoscopy as a procedure to detect melanoma in their patients.

For categorization, the shape, color, and Pyramid Histogram of Oriented Gradients (PHOG) properties are provided in the literature. This method [3] proposes an approach that automatically identifies melanoma using the ABCD rule. Gabor filters are used in the preprocessing step to identify the hair and geodesic contours are used to identify the borders. The strategy used by Kasmi et al. [3] incorporates both conventional and cutting-edge techniques. To extract the properties of ABCD attributes, algorithms are used. This method’s disadvantage is that the performance needed to be raised. Convolutional neural network (CNN) use has been suggested by Zhang et al. [7] for the categorization of skin cancer. This approach has produced positive outcomes. Color characteristics and an instance-based learning method were utilized by Pereira et al. [4], achieving an accuracy of 61.7 percent. Deep Convolutional Neural Networks for classification have been proposed by Harangi et al. [5]. For classification, they used convolutional neural networks (CNNs). The results of various deep network topologies were combined. However, this proposed method had a flaw in that it required more data for training and took a long time due to the multiple layers involved [8]. The idea of melanoma detection using image processing techniques was put forth by Garg et al. [16]. Dermoscopy and light microscopy were employed in the procedure. However, the automatic diagnosis technique was less expensive. To reduce the additional noise in the image, preprocessing was done. After that, segmentation was applied. Mukherjee et al. [17] suggested a metaheuristic technique that is inspired by nature and finds ideal solutions quickly and effectively. Multi-Layer Perceptron (MLP) classifies melanoma, achieving an accuracy of more than 91 percent. When compared to other works in the literature, it produces positive outcomes. The parameters of the optimization approaches are examined in a two-dimensional space. The optimization process for this method takes a long time. By combining the features from the various methodologies, Hagerty et al. [40] suggested a strategy that blends traditional image processing and deep learning. Deep learning and traditional image processing were the two methods they suggested. For classification, deep learning with the ResNet-50 is utilized. For prediction, logistic regression is used. It identifies only color features when there is a lesion, which is a drawback. According to Kaur et al. [41], dermoscopy picture classification might be accomplished by combining texture and color data. The texture is extracted using a local binary pattern (LBP), which abstracts a histogram and scale-adaptive patterns from each pixel. Their histograms were HSV ones. For categorization, concatenated features are provided. Although this method produced good results, it has the disadvantage that the LBP does not collect border information. For more accuracy than current methods, the suggested Sobel Directional Pattern (SDP) for feature extraction integrates key characteristics of skin melanoma, such as texture, color, and boundary information, into a feature vector. Compared to other approaches, this model uses stacked Restricted Boltzmann Machines (SRBMs) that are quicker and more precise. When dealing with unbalanced datasets, the stacked RBM also produces good results. The currently used feature descriptors either record edge or texture information. The suggested method successfully captures edge, texture, and color information.

The pixels of biomedical images are used by computer vision to categorize the different forms of skin cancer. Shape, borders/edges, texture, color, and other details are estimated using the dermoscopy images individually in the literature. In the present efforts, Support Vector Machines (SVMs) are primarily utilized [42]. These can only classify things in binary. When utilized as a one vs. one model for multiclassification, this method takes more time. When there are fewer samples available, Deep Neural Networks (DNN) [43] are employed in some works, which leads to the overfitting issue. There is a lack of generalization and poor classification accuracy because of images with lower resolution and the differences in dermoscopy of the images. In order to emphasize the importance of the work that we are about to discuss, we have made a list of the contributions that the current study has made, which are as follows:

- A hybrid AI-based framework based on stacked Restricted Boltzmann Machines and Sobel Directional Patterns is proposed for Skin Melanoma Prediction;
- The Sobel Directional Pattern (SDP) is a new method for feature extraction that combines texture, color, and edge data into a single feature vector using a Sobel filter. The presented image preprocessing phase helps in removing the noise, enhancing the image quality by stitching the histogram, and removing the hairs from the images. This process also removes the random noise that is introduced to an image during its acquisition;
- An automatic semantic segmentation using FCCNs is provided for extracting the ROIs from the skin melanoma lesions. Stacked RBMs are used to accurately classify the segmented cutaneous lesions;
- Preprocessing is performed to reduce the hairs, and the need to align images into random datasets during image acquisition is eliminated;
- The newly discovered and adapted Sobel filter-based Sobel Directional Pattern (SDP) extracts features and mixes texture, color, and edge data into a single feature vector.

The suggested method improves image quality by removing hairs through preprocessing. The suggested SDP also eliminates noisy data, improving accuracy. The most discriminating data are determined as a feature vector utilizing SDP. The SDP feature extraction approach is used in this method to encode the color, edges, and op-ponent color and texture data as a feature vector. For categorization, a stacked RBM is employed. The suggested model is faster and more accurate than the ones currently in use. For comparing grayscale and color texture features, the SDP operator is created as a combined color-texture operator. Accuracy is enhanced by the use of both color and texture elements. The term “Opponent colors” refers to all pairs of color channels. Here, each color channel receives a distinct application of the directional pattern produced by the Kirsch masks operator. For further calculations, only the maximum responses are employed.

This eliminates the erratic noise that was picked up during image capture. Each pair of color channels is also used to derive the patterns of the opposition. A neighborhood’s center pixel and surrounding pixels are drawn from different channels. Feature extraction, preprocessing, and classification are the steps in the proposed system’s framework for classifying skin cancer. With its greater processing power and quicker learning, the stacked RBM in the Deep Belief Network achieves accurate prediction in a shorter period. For unbalanced datasets, the stacked RBM in the Deep Belief Network also performs well. 

The suggested methodology is described in Section 3, which also covers the capture of images, the preprocessing method, feature extraction with SDP, and classification with RBM. Section 4 of the report discusses the experimental findings. The proposed strategy is also contrasted with other cutting-edge methods in Section 4.

## 2. Materials and Methods

### 2.1. Skin Image Datasets

**Figure 1:**
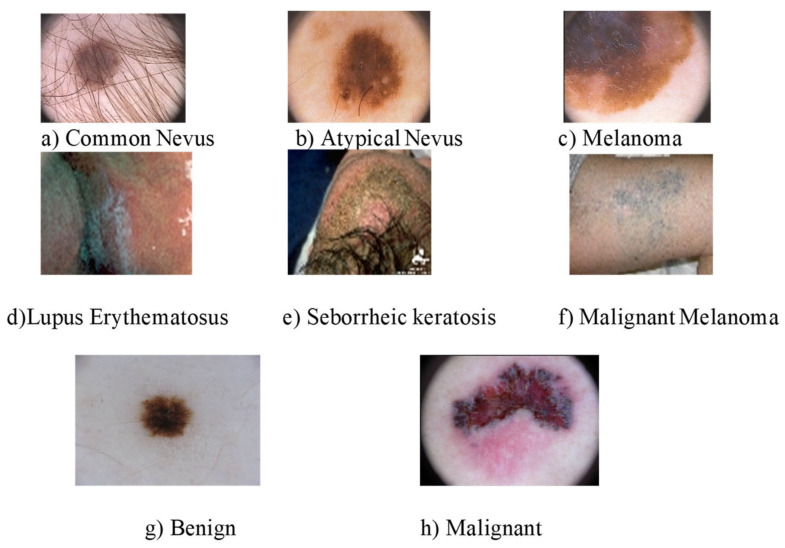
Samples of utilized images in different datasets.

The data that were used in this study were gathered from five different public datasets including PH2 [44], ISIC 2016 [45], ISIC 2017 [45], DermIS (https://www.dermis.net/doia/ accessed on 1 December 2022), and DermNet NZ (https://dermnetnz.org/image-library accessed on 1 December 2022). The images in PH2 are divided into melanoma and non-melanoma categories. Both cancerous (melanoma) and noncancerous (benign) skin lesions are represented in the 2016 dataset collected by the International Skin Imaging Collaboration (ISIC). There are a total of 1279 images available in the ISIC 2016 dataset, including 900 training images and 379 test images. Both the training and testing sets include ground truth data that indicate whether or not each lesion is cancerous. Out of a total of 2600 images, 2000 are used for training and 600 are used for testing in the ISIC 2017 dataset. Melanoma, seborrheic keratosis, and nevus are the classes that are represented, and the ground truth and patient metadata are included in the training and testing sets. The Dermnet Skin Disease Atlas has tagged 23,000 images on the platform. Images from 23 classes are included in this dataset. The tests use three kinds of disorders from Dermnet, including Molluscum contagiosum, Seborrheic Keratosis, and Metastatic Melanoma. Melanoma, Seborrheic keratosis, and lupus erythematosus are the three skin conditions from DermIS that are taken into consideration for the tests. Figure 1 depicts several sample images.

### 2.2. Proposed End-to-End CAD Framework for Skin Lesions

**Figure 2:**
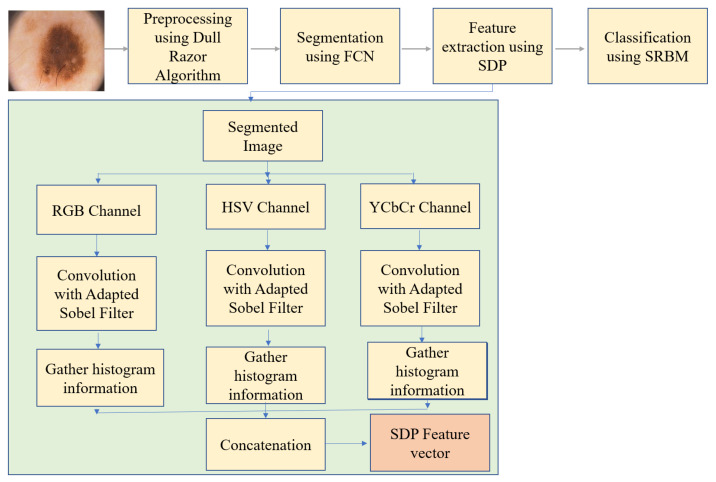
Proposed framework of the current study.

This research presents an end-to-end system for the diagnosis of skin melanomas using SDP and stacked RBM algorithms. Major elements of the introduced framework are shown in Figure 2. Preprocessing techniques are used on captured images to improve their quality, to align images, and to get rid of distracting hairs. Then, in order to extract informative Regions of Interest (ROIs), a semantic segmentation technique based on FCNN is created. This study introduces a comprehensive framework for identifying skin melanomas with the use of SDP and stacked RBMs. Once the image has been removed of its noise, it is put into the SDP feature extraction algorithm, which chooses just the maximum response data produced by the customized Sobel filter-based masks. This technique can be used to isolate subtle color differences, textures, and outlines of fine lines. If you use one of the alternative filters, you will get a significant amount of extra noise around the edges. The resulting feature vectors are then used as input for classification by the stacked RBM. According to the attributes extracted by SDP, the images are then classified into several skin cancer classes.

#### 2.2.1. Image Preprocessing

**Figure 3:**
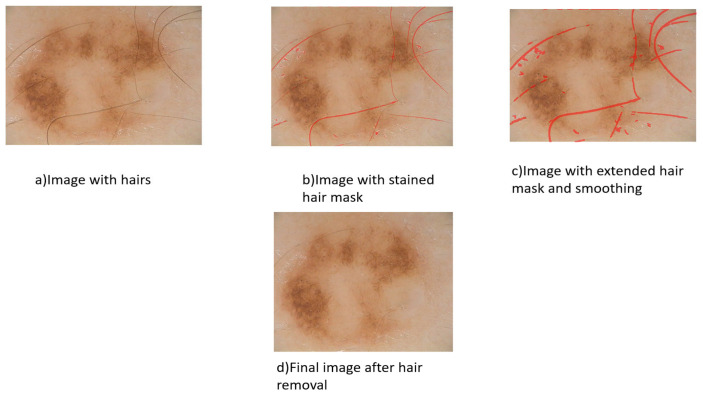
Stages of hair removal of images after applying the DullRazor algorithm.

The first thing that has to be done is some preprocessing on the images in order to find and get rid of any hairs that are visible on the skin. It is possible that classification errors will occur if there are hairs present in the skin imaging data. As a consequence of this, the procedure of removing hairs using DullRazor is utilized at this stage of preparation. It is able to do this by carrying out an activity called broad grayscale morphological closing, which allows it to recognize the locations of dark hair. It does this by alternating the pixels that have been validated with bilinear interpolation and confirming that the structure of the hair pixel outline is thin and extended. An adaptive median filter is applied in order to smooth out the pixels that represent the changed hair. In order to generate a hair mask, the images including hair must first undergo preprocessing. As can be seen in Figure 3, images are preprocessed to eliminate hair by making use of an algorithm called DullRazor [46]. Because the images are not all the exact size, each one of them has to have its dimensions adjusted. Each image has been scaled down to the exact dimensions, which are 760 pixels wide by 570 pixels tall. Images were selected from the Pedro Hispano Hospital (PH2) dataset in accordance with their respective average sizes and are given after hair removal in Figure 3.

#### 2.2.2. Image Segmentation

**Figure 4:**
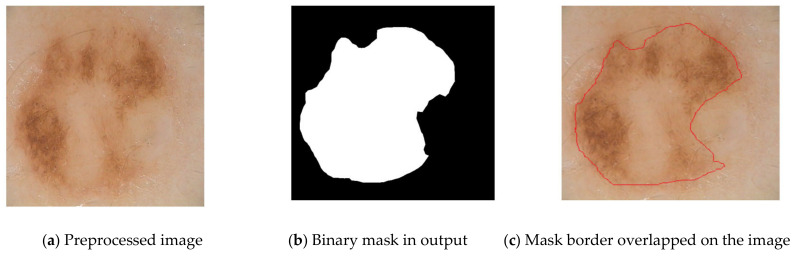
An example for a segmented skin image.

Using the segmentation method, the skin lesion is divided into its subcomponents [47]. Figure 4 presents the images after they have been segmented. Morphological operations can be performed on the data obtained from skin imaging to help locate skin lesions. It is also possible to obtain the image’s complementary black and white version. There is a clear distinction between the background and the skin lesion. It has been shown that the strategy of increasing regions is particularly helpful for detecting skin. The region-growing strategy is another method that can be utilized in the process of extracting the lesion from the images of the skin. In this proposed work, FCCNs are utilized for segmentation purposes. Fully Connected Convolutional Networks are a form of architecture that are utilized in semantic segmentation. They exclusively employ convolution, pooling, and up sampling as their locally connected layers to build their models. FCCNs give each pixel a classification in order to achieve a certain level of semantic segmentation for images. Figure 4 depicts an example of a segmented image.

#### 2.2.3. Feature Extraction 

Dermatologists define melanoma using the ABCD rule [3], which they utilize to evaluate skin lesions. Images are examined for irregular borders, asymmetry, and uneven distribution. DermIS, PH2, ISIC 2016, and ISIC 2017 are the datasets that were utilized. For categorizing the images of skin cancer in this study, novel SDP and stacked RBM are used. It is no longer necessary to use different computational methods to extract information about texture, color, and edges from skin cancer images.

The method of mining the essential data from the obtainable raw images is called feature extraction. When used as input by a machine learning algorithm, the collected features have to be non-redundant and produce good outcomes. Using the feature extraction method, the dataset’s images are condensed into a tiny feature vector. The suggested feature extraction method uses a minimal number of computations to merge the edge, color, and texture data into a feature vector. The skin pictures with the lesion are given measurable information via the proposed SDP approach. This method can be utilized as a skin cancer early detection tool. By doing this, more painful procedures of skin cancer diagnosis are avoided. Digital images are used in this manner. The ABCD rule of dermoscopy is the foundation for the feature extraction techniques seen in the literature. ABCD stands for Asymmetry, Border, Color, and Diameter. Dermatologists utilize these characteristics to categorize melanomas. Asymmetry, irregular boundaries, hue, uneven distribution, and a diameter larger than 6 mm are the characteristics examined:

▪ **Asymmetrical Shape:** Lesions are uneven or asymmetrical in shape, denoted by the letter A. Other moles are regular and benign;
▪ **Border:** The borders of non-cancerous moles are even and smooth. The borders of the melanoma lesions are erratic;
▪ **Color:** More hues including blue, black, brown, and tan are found in melanoma. The uneven distribution of hue is an indication of possible melanoma. Moles that are benign only come in one hue of brown;
▪ **Diameter:** Lesions with melanoma have a diameter of more than 6 millimeters.

The suggested SDP takes the skin cancer images and extracts the color, texture, and edge-based properties. In the suggested study, the characteristics can be taken from the various color spaces, such as RGB, HSV, and YCbCr, and provided as input to the classifier for melanoma prediction [45]. The RGB, HSV, and YCbCr color spaces all allow for effective color discrimination. The RGB, HSV, and YCbCr space color and texture information can be extracted using the SDP operator. The SDP operator is applied separately to each color channel in an HSV image in this proposed approach as follows. The various color channel pairs are employed to collect various color patterns. Numerous color channels are used to choose the epicenter and location pixels. In SDP_H,S_, the middle pixel in a 3×3 region is designed with the pixel at center position from H and the nearby pixels from S. In the H-channel image, H_c,d_ is the pixel at the center and H_c,d_, H_c+1,d_, H_c−1,d_, H_c−1,d+1_, H_c,d+1_, H_c+1,d+1_, H_c−1,d−1_, H_c,d−1_, and H_c+1,d−1_ are the eight adjacent pixels in a block. In the S-channel image, S_c,d_ is the pixel at the center and S_c,d_, S_c+1,d_, S_c−1,d_, S_c−1,d+1_, S_c,d+1_, S_c+1,d+1_, S_c−1,d−1_, S_c,d−1_, and S_c+1,d−1_ are the pixels present at the eight adjacent sides in a block. In the V-channel image, V_c,d_ is the pixel at the center and V_c,d_, V_c+1,d_,V_c−1,d_, V_c−1,d+1_, V_c,d+1_, V_c+1,d+1_, V_c−1,d−1_, V_c,d−1_, and V_c+1,d−1_ are the pixels present at the eight adjacent sides in a block. SDP_H,H_, SDP_S,S_, SDP_V,V_, SDP_H,S_, SDP_H,V_, and SDP_S,V_ are the combined channel images. Here, each 3 × 3 block is formed with the subsequent equations:

SDPH,H (p,q,θ) = <center (H_c,d_), neighbors (H_c + i,d + j_) >, SDPS,S (p,q,θ) = <center (S_c,d_), neighbors (S_c + i,d + j_) >, SDPV,V (p,q,θ) = <center (V_c,d_), neighbors (V_c + i,d + j_) >, SDPH,S (p,q,θ) = <center (H_c,d_), neighbors (S_c + i,d + j_) >, SDPH,V (p,q,θ) = <center (H_c,d_), neighbors (V_c + i,d + j_) >, SDPS,V (p,q,θ) = <center (S_c,d_), neighbors (V_c + i,d + j_) >, (1)

where, for a 3 × 3 block 1 ≥ p ≤ 4 and 1 ≥ q ≤ T, T is the total number of blocks in each image.

(2) $$\left(i,j\right)=\left\{\begin{array}{c} i=1,j=1\;\;if\;\theta =45{}^{\circ}\\ i=1,j=-1\;\;if\;\theta =315{}^{\circ}\\ \;i=-1,j=-1\;\;if\;\theta =225{}^{\circ}\\ i=-1,j=1\;\;if\;\theta =135{}^{\circ}\\ i=0,j=-1\;\;if\;\theta =270{}^{\circ}\\ i=0,j=1\;\;if\;\theta =90{}^{\circ}\\ i=1,j=0\;\;if\;\theta =360{}^{\circ}\\ i=-1,j=0\;\;if\;\theta =180{}^{\circ} \end{array}\right..$$

**Figure 5:**
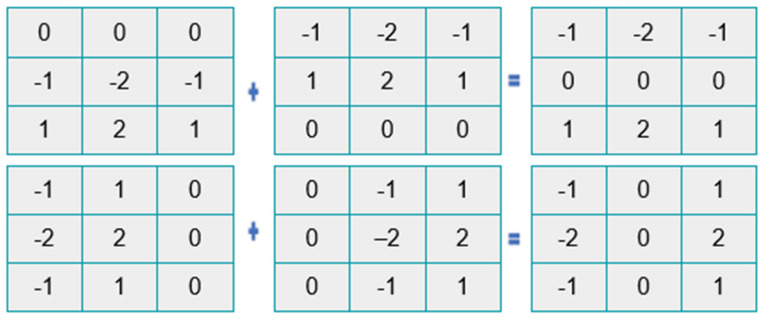
The adapted Sobel representation.

**Figure 6:**
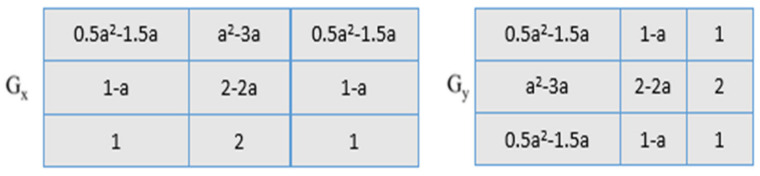
Left fractional-order Sobel.

**Figure 7:**
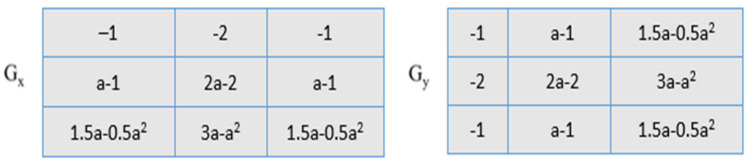
Right fractional-order Sobel.

The greatest value obtained after the convolution of the mask and the picture is used to calculate the edge magnitude. The mask that creates the greatest magnitude determines the orientation of the edge. The different channel combinations of the skin images (combined channel images) SDP_H,H_, SDP_S,S_, SDP_V,V_, SDP_H,S_, SDP_H,V_, and SDP_S,V_ are provided to SDP as input to create the feature vector. Three stages make up the feature extraction procedure for each channel image: compass mask filtering of skin images, code image generation based on the maximum response, and feature vector construction. The feature extraction procedure is also applied to the other color channels, such as RGB and YCbCr [19,47,48]. After taking into account the preprocessed images, fractional-order Sobel masks [49] are combined with them to analyze the pattern using the proposed SDP. The fractional-order masks, have shown in Figure 5, Figure 6 and Figure 7.

The customized Sobel illustration can be made by altering the design of the *G_a_* and *G_b_* parts of the special Sobel filter. Both the x- and y-axes contain the gradient-based parts of the integer-order Sobel operator. The components’ differential form is denoted by:

(3) $$G_{a}=2\left(\frac{\partial f\left(a+1,b-1\right)}{\partial a}+2\frac{\partial f\left(a+1,b\right)}{\partial a}+\frac{\partial f\left(a+1,b+1\right)}{\partial a}\right)$$

(4) $$G_{b}=2\left(\frac{\partial f\left(a-1,b+1\right)}{\partial b}+2\frac{\partial f\left(a,b+1\right)}{\partial b}+\frac{\partial f\left(a+1,b+1\right)}{\partial b}\right)$$

In the actual Sobel filter, the *G_a_* and *G_b_* components are reorganized as in Figure 5 and Figure 7. The differential form of the effective Sobel filter is determined from the gradient operator in the equation above, and it is then translated to the fractional-order domain. Two novel masks, the left fractional Sobel mask and the right fractional mask, are produced using the Grunwald–Letnikov (GL) fractional-order differential operator. The left GL derivative is signified as:

(5) Mag (∇αf)Stαf(t)=1 mαm→0+lim∑j=0∞−1jαjft−jm=Ga+Ga.

To attain a symmetric filter, the GL operator is functional on the updated Sobel filter.

The right GL derivatives are signified as:

(6) Stαf(t)=1 mαm→0+lim∑j=0∞−1jαjft+jm.

When applied to the images, these modified fractional-order Sobel masks assist in extracting the thin edges as opposed to the thick edges produced using the integer-order Sobel masks. Some responses are created after convolution of each pixel with the adapted fractional-order Sobel masks/filters. Using the maximum intensity value among the responses obtained for each pixel, a code image is created. The DOG filtering method is used to remove noise from the code image. After that, the code image is divided into smaller grids, and the histogram is measured for each grid. The final feature vector is produced after computing the feature vector for each grid, grouping it, and then combining it. In order to get a better presentation compared to the standard feature descriptors, a multi-scale feature descriptor with rotation invariance and low complexity is suggested in this work as SDP. 

#### 2.2.4. Enhanced Sobel Masks Representation

The cropped images are convoluted with the fractional-order Sobel masks. As the value α of the fractional mask varies, the convoluted output differs. The fractional-order filters are highly sensitive to variations in edge compared to the normal Sobel masks. These fractional-order masks, as shown in Figure 5, Figure 6 and Figure 7, help to capture more details regarding the texture, resulting in high classification accuracy of the human emotions. The values of α range from 0.1 to 1 and λ ranges from 1 to 5 (λ = {3.5, 7, 14, 28, 56}). The value of α = 1 results in a conventional Sobel mask. 

The fractional-order Sobel masks are convoluted with the cropped images. The output of the convolved algorithm differs depending on the value of the fractional mask. Compared to the standard Sobel masks, the fractional-order filters are much more sensitive to edge alterations. These fractional-order masks, as displayed in Figure 3 and Figure 4, aid in capturing additional texture-related features and have a high classification accuracy for human emotions. Furthermore, α have values ranging from 0.1 to 1 and λ from 1 to 5, respectively (λ = 3.5, 7, 14, 28, 56). A conventional Sobel mask is produced when α = 1. 

To determine the best value for, experiments we have conducted using a range of values between α = 0.1 and α = 1, and the dataset images are classified using a K-Neural Network (KNN) classifier. The left and right fractional Sobel masks are combined with the segmented images. The segmented images are convolved with the fractional Sobel masks that are suggested in this paper. The highest response value possible is chosen since each pixel receives four responses. The answers are {$S_{\theta_{0}},S_{\theta_{1}}\ldots .S_{\theta_{7}}\}.\;$ The following is the formula for choosing the Maximum Response (MR):

(7) $$\mathrm{MR}\left(x,y\right)=\max(S_{i}\left(a,b\right)|0\leq \;i\;\leq 4).$$

Here, $T_{\theta }{}_{i}\left(a,b\right)$ denotes the response attained at an exact pixel position $\left(a,b\right)$. Then, the Difference of Gaussian (DOG) filter is applied on the MR image as follows:

(8) $$D=\mathrm{DOG}\left(\left(a,b\right);\sigma_{1},\sigma_{2}\right)=\frac{1}{2{\pi \sigma }_{1}{}^{2}}e^{-\frac{a^{2}+b^{2}}{2\sigma_{1}{}^{2}}}-\frac{1}{2{\pi \sigma }_{2}{}^{2}}e^{-\frac{a^{2}+b^{2}}{2\sigma_{2}{}^{2}}}\;,$$

where ${\;\sigma }_{1}$ is the standard deviation that is higher than $\sigma_{2}$.

(9) $$f\left(x,y\right)=M\left(x,y\right)*D.$$

**Figure 8:**
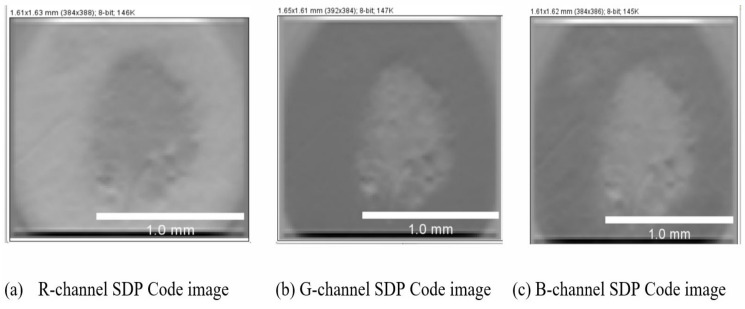
Sobel code images of the PH2 dataset; the size of the images are 238.2 pixels/mm.

Convolution of the response images and the DOG filter help to improve classification accuracy by removing random noise and sharpening the edges. Figure 8 represents the code images created as a result of SDP. The code images for R, G, and B channels, as in Figure 8a–c, signify both the textural and edge-based information gathered from images and also indicate the portions of the image that result in effective classification. The grids made over the code image are used to construct the histogram. The feature vectors are fed to a stack of Restricted Boltzmann Machines for prediction.

#### 2.2.5. Skin ROIs Classification 

In this work, SRBM is utilized for classification purposes. When compared to stacked Restricted Boltzmann Machines, SRBM is distinguished by the fact that it prohibits lateral connections inside a layer in order to make analysis simpler. On the other hand, the stacked Boltzmann method combines a supervised top layer for class recognition with an unsupervised three-layer network that has symmetric weights. This results in a hybrid model. The stacked Boltzmann method is applied in the comprehension of natural languages, the retrieval of documents, the creation of images, and the classification of these. These functions can be trained through either unsupervised preliminary training or through supervised fine-tuning. In contrast to the top layer, which is symmetric but without any direction, the RBM connecting layer is asymmetric and bidirectional. The restricted Boltzmann connection, which brings together two separate networks into a single entity, is composed of three layers with asymmetric weights. Both stacked Boltzmann Machines and Restricted Boltzmann Machines have this in common: the neural building pieces that make up their networks are composed of stochastic binary Hopfield neurons. Gibb’s probability measure takes into account the energy from Restricted Boltzmann as well as RBM; Boltzmann is analogous to RBM. There is no back propagation in the restricted Boltzmann train, which processes one layer at a time, makes a three-segment pass to an approximation of the equilibrium state, and does not use back propagation. The Restricted Boltzmann method performs pre-training for classification and recognition using supervised and unsupervised training on different RBMs. 

### 2.3. Evaluation Methods

For the experiments, ten-fold cross-validation is used. The proposed strategy is compared to other cutting-edge techniques using the following metrics. Regarding accuracy, Harmonic Mean (HM), positive predictive rate (pp), sensitivity, specificity, and F-score [32,50,51,52,53,54] are the measures used to assess performance and they are defined by Equations (10)–(15). Sensitivity refers to the percentage of true positives that have been accurately detected or the number of individuals who have been appropriately identified as having melanoma. Accuracy refers to the proportion of a sample out of the complete population that has been correctly classified. Specificity is evidence that the patients concerned do not relate to any kind of skin melanoma. The percentage of tests that correctly identify a patient is referred to as the positive predictive rate. The F1-score represents the weighted mean harmonic average. The definition of these Metrics is represented by true positives (TP), true negatives (TN), false positives (FP), and false negatives (FN).

(10) $$\mathrm{Accuracy}\;\left(\mathrm{ACC}\right)=\frac{\mathrm{TP}+\mathrm{TN}}{\mathrm{TP}+\mathrm{TN}+\mathrm{FP}+\mathrm{FN}},$$

(11) $$\mathrm{Specificity}\;\left(\mathrm{SP}\right)=\frac{\mathrm{TN}}{\mathrm{TN}+\mathrm{FP}},$$

(12) $$\mathrm{Positive}\;\mathrm{Predictive}\;\mathrm{Rate}\;\left(\mathrm{pp}\right)=\frac{\mathrm{TP}}{\mathrm{TP}+\mathrm{FP}},$$

(13) $$\mathrm{Sensitivity}\;\left(\mathrm{SE}\right)=1-\frac{\mathrm{TP}}{\mathrm{FN}}\;,$$

(14) $$F-\mathrm{Score}=\frac{2*\mathrm{Sensitivity}*\mathrm{pp}\;}{\mathrm{Sensitivity}+\mathrm{pp}},$$

(15) $$\mathrm{Harmonic}\;\mathrm{Mean}\;\left(\mathrm{HM}\right)=\frac{2*\mathrm{Sensitivity}*\mathrm{Specifity}}{\mathrm{Sensitivity}+\mathrm{Specifity}}\;.$$

### 2.4. Model Training and Hyperparameters

Cross-validation is an iterative method for preventing the practice of overfitting in predictive methods. Each individual part of the dataset had to be separated out into its own section. In order to carry out a standard K-fold cross-validation, the data had to be segmented into k-folds first. Then, while we were repeatedly retraining the algorithm on k−1 folds, we included the remaining holdout fold as the test set. In this research we used k cross-validation on to 10-fold.

The hyper-parameter values that are used in RBM are detailed in Table 1. Because it is more capable of generalization than the conventional DNN, the suggested model makes use of a stacked RBM in order to classify the images of skin cancer. The particle swarm optimization approach is utilized in order to fine-tune the RBM hyper-parameters. The persistent contrastive divergence is utilized in order to generate a rough estimate of the likelihood gradient. Only the very first and very last repetitions of the convergence process are successful when the Markov chain has a low mixing. After that, an RBM model constructed with each training sample is used. Following the completion of each Gibbs iteration, the model is reconstructed, and the aforementioned method is then repeated for each epoch. 

## 3. Results 

In order to conduct our investigation, we relied on both a graphics processing unit (GPU) and a central processing unit (CPU) developed by Intel. The algorithms were implemented using version 7.12 of MATLAB. The different sets of images acquired from the databases do not overlap with one another in any way. On five different datasets, we conducted an analysis to determine how well the newly developed framework, Hybrid SDP, and stacked RBM, classified skin lesions as either cancerous or benign. This analysis was based on the values that were generated by the performance metrics. In addition, the effectiveness of the classification system was evaluated alongside more traditional techniques, such as the support vector machine (SVM), Gradient Boosting (GB), and Random Forest (RF). Table 2, Table 3, Table 4, Table 5, Table 6, Table 7, Table 8, Table 9, Table 10 and Table 11 present the findings of the experiments performed on the five datasets. For specificity and sensitivity, the average of the values is reached. Compared to Support Vector Machine (SVM) and Gradient Boosting (GB), the random forest classifier produces better results. However, compared to the GB, Random Forest (RF), and SVM, the stacked RBM used in the suggested approach produces the greatest results. When distinguishing melanoma from dysplastic nevi, the suggested method produces the best results. The texture, edge, and color information in the proposed study helps all of the classifiers achieve better outcomes by producing greater SE and SP. There is a 0.2 percent increase in sensitivity for ISIC 2016 and ISIC 2017 when utilizing stacked RBM. The proposed technique performs well for the Dermnet and DermIS datasets. The images in the collection were captured using a variety of tools and under a variety of lighting situations. SDP performs well in comparison to the other feature descriptors since it is immune to variations in illumination. Tables denote specificity as SP, sensitivity as SE, Positive predictive rate as PP, Harmonic Mean as HM, and accuracy as ACC. Three channel images were created from the original photographs. Six possible combinations of the three channel images were gained for the SDP algorithm. Using stacked RBM, the feature vectors were predicted, and the outcomes were then obtained. Three channels made up the image. The visuals for the red, green, and blue channels were then produced. Then, several channel combinations were made, and the SDP method extracted the final feature vector from the code image that SDP generated. Utilizing histograms, the code image produced by the SDP technique was used to construct feature vectors. When the photos from the PH2 dataset were utilized for the tests, the confusion matrix shown in Table 2 was created. The overall positive rate for common nevi was found as 100%. Atypical nevi have a TPR of 99%, while melanoma have a 100% TPR. The suggested SDP algorithm is compared to the other feature extraction methods in the literature in Table 3. The findings show that the proposed method obtains a high classification accuracy of 99.8%. The RBF kernel was utilized for categorization using a Least Square Support Vector Machine (LS-SVM), SVM, and Extreme Learning Machine (ELM). There are 1 to 50 hidden layers in a multi-layer perceptron, which uses the Levenberg–Marquardt optimization. The TPR for the benign group is 100%, whereas the TPR for melanoma is 94%. The confusion matrix and the categorization outcomes for the photos from the ISIC 2016 dataset are shown in Table 4 and Table 5. Compared to LBP (Local Binary Pattern), CLDP (Color Local Directional Pattern) [18,55,56,57,58] has the highest accuracy. The accuracy of 97.2 percent shows that, compared to GLCM [59], LBP more accurately captures the texture of skin cancer images [18]. In comparison to LBP, CLDP achieves higher sensitivity and specificity. When used in conjunction with stacked RBM, the suggested method for SDP yields the best accuracy. It has a 99.8% accuracy rate for the PH2 dataset, which is the best. The achieved sensitivity is 98.8 and the achieved specificity is 99.6. The HM is 99.4, the PP is 99.6, and the F-Score is 99.6. By choosing only the most relevant answer information, the proposed SDP eliminates all the noisy information and outperforms the other existing descriptors in terms of accuracy.

The ISIC 2016 dataset’s poor representation of the lesion’s size and location from the PH2 dataset leads to incorrect classifications of the lesions. For the ISIC 2016 datasets, the proposed technique achieves specificity of 92.5 and sensitivity of 95.7 in Table 5. When compared to the outcomes obtained by LBP and CLDP, the suggested method’s classification accuracy is 96.5 percent, which is high. The ISIC 2017 dataset’s confusion matrix is shown in Table 6. The TPR for benign types is 96%, whereas the TPR for melanoma types is 97%. The results obtained from utilizing the photos from the ISIC 2017 dataset are shown in Table 7.

The classification outcomes for ISIC 2017 are shown in Table 7. For the ISIC 2017 datasets, the suggested method yields a specificity of 98.5 and a sensitivity of 99.9. Comparing the suggested method’s classification accuracy to those obtained by LBP and GLCM [27], it is high at 95.5 percent. In order to calculate the AUC, the average of the cross-validation results obtained for each dataset is calculated.

**Figure 9:**
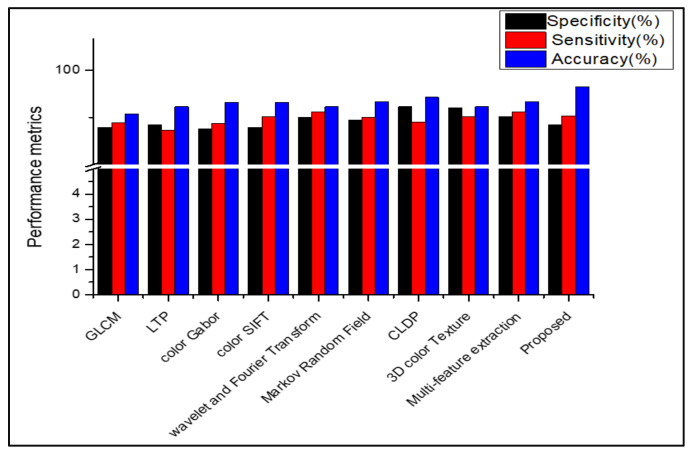
Comparison of performance using various feature descriptors in PH2.

**Figure 10:**
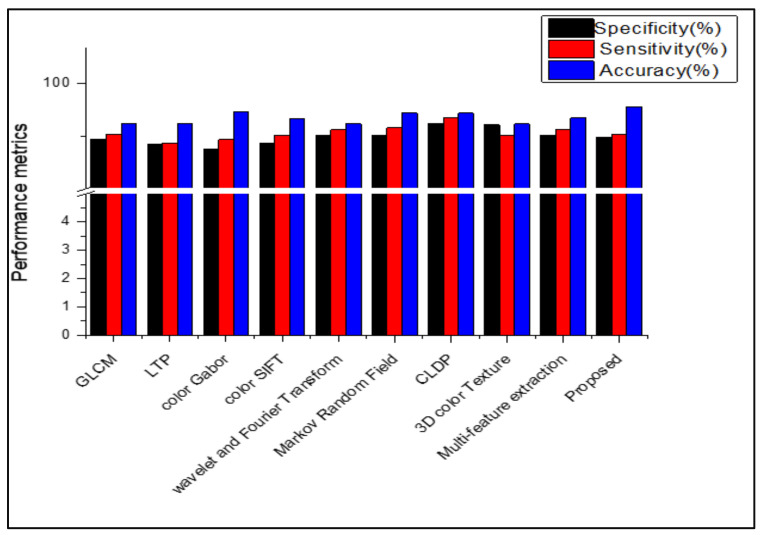
Comparison of performance using various features in ISIC 2016.

**Figure 11:**
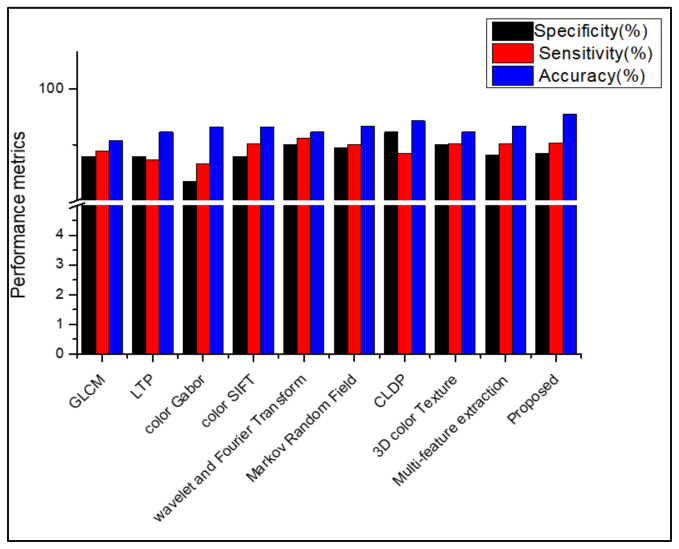
Comparison of performance using various features in ISIC 2017.

**Figure 12:**
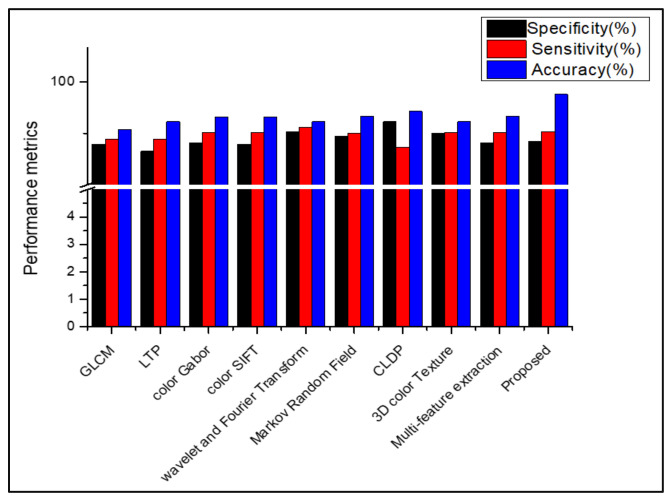
Comparison of performance using various features in DermIS.

**Figure 13:**
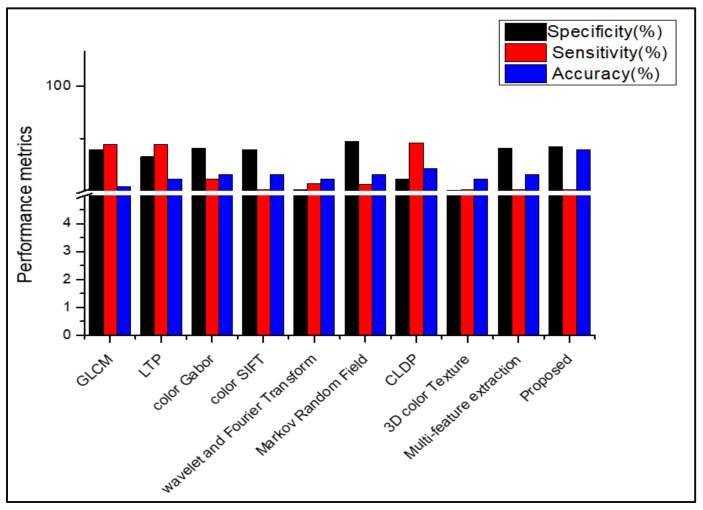
Comparison of performance using various features in Dermnet.

Table 8, Table 9, Table 10 and Table 11 show that, when the suggested work is used to classify the datasets Dermnet and DermIS, a high level of classification accuracy is attained. For unbalanced datasets, the stacked RBM in the Deep Belief Network also performs well. The SDP feature and the stacked RBM in the DBN are used to attain the highest classification. Several descriptors from the literature are used in place of the proposed feature descriptor SDP, and the results are equated for all those datasets, as shown in Figure 9, Figure 10, Figure 11, Figure 12 and Figure 13. Comparing the proposed feature descriptor SDP to all existing feature extraction methods in the literature, it obtains good performance because of its capacity to capture the edge information, histogram information from rival colors, and texture information. SDP more successfully recovers the spatial data of the texture, edges, and opponent color information while also removing noise. Other feature descriptors, such as LTP, Color SIFT, Gradient information, CLDP, Color Gabor wavelet, and multi-feature extraction, do not completely reduce the noise [8,18,55,56,58]. In contrast to LBP and additional descriptors used in the existing methods for the diagnosis of skin lesions, SDP is likewise insensitive to changes in light.

Differential diagnosis of melanoma includes other pigmented lesions such as basal cell carcinoma (ISIC 2019), Bowen disease, Actinic keratosis, and squamous cell carcinoma, which are also analyzed from the ISIC 2019 dataset from images that achieve a TPR rate of 91% for basal cell carcinoma, 83% for Bowen disease, and 62% for squamous cell carcinoma, as in Supplementary Table S1. The proposed approach is compared with the other state-of-the-art approaches in Supplementary Table S2. The effectiveness of various cutting-edge techniques is measured against the outcomes shown in Supplementary Table S2 to compare performance. For the datasets used in the research for the prediction of the images using depth and 3-D shape, Satheesha et al. [60] have suggested a 3D skin lesion reconstruction. Bi et al. [61] have proposed a method that uses a multiscale lesion-based portrayal and classification utilizing a combined reverse approach. They used photos that had been preprocessed to change the contrast. Waheed et al. [62] used color and texture factors as well as contrast adjustments to categorize melanoma. Gutman et al. [45] analyzed skin lesions with a 91.6 percent accuracy rate. Lopez et al. [63] used CNN [32] to analyze spatial domain, but the suggested method makes use of color, structural, and textural data to produce accurate classification findings. Matsunaga et al. [64] classified data using DNN. Deep learning methods achieve good accuracy for skin cancer classification [65,66]. By using the Newton–Raphson approach, Khan et al. [67] have presented a region-based convolutional neural network. Using the DermIS dataset, DNN was implemented by Bajwa et al. [68]. Using the Dermnet dataset, Rajinikanth et al. [69] employed the Bat algorithm. As shown in Table S2, the suggested method using stacked RBM in the Deep Belief Network delivers higher prediction accuracy compared to CNN, DNN, and other cutting-edge methods.

## 4. Limitations and Future Work 

The feature values acquired using feature extraction algorithms are frequently dispersed when the images have diverse zooming settings, variable lighting, and different resolutions. In the suggested method, a normalization strategy is used to get around this issue. The information required to complete the normalization procedure can be found in [70]. The classification technique’s stacked RBM generates a reliable and ideal prediction. When numerous boundaries or ambiguous borders are present in the images, the classification fails. In those circumstances, crucial details are lost, which causes the accuracy to drop, as demonstrated by the trials. Principal Component Analysis can be used to reduce the feature vector’s rise in dimension. Even in unbalanced datasets, the stacked RBM in the DBN produces good results. Even images with poor quality and little contrast produce excellent results. There are not many works in the literature that discuss the categorization of melanoma and dysplastic lesions. The proposed effort produces superior outcomes in this study, similar to those seen in the results presented for SE and SP. SDP is thus perfectly suited for use in real-time applications because of its high accuracy and low processing complexity. The suggested SDP enhances the image and eliminates any potential noise using Difference of Gaussian filters. The retrieved features make this feature extraction method superior to those currently in use, in addition to being scale- and rotation-invariant. However, the Sobel operator’s primary shortcoming is its signal-to-noise ratio. As noise levels increase, the gradient magnitude of the edges becomes smaller, which could lead to some incorrect results. The use of derivatives based on fractional orders on better edge detection methods will be used in the future to address this SDP restriction. Our proposed model could be implemented by doctors and dermatologists for clinical use. Finally, many challenges and ideas from other domains such as NLP and image processing can be investigated by applying hybrid models in future. The proposed work will be improved by including other fractional-order-based derivatives to enhance the performance of these categories in future works. Furthermore, it is possible to adapt the proposed algorithms to identify features that are included in a seven-point checklist with pattern analysis for dermoscopic equivocal melanocytic lesions while applying other fractional-order-based derivatives with some other better edge detection methods in future works. 

## 5. Conclusions

SDP results from the interaction of color, edge, and texture elements, which dynamically acquires the structural qualities of the image and increases data discrimination. The results show that the suggested methodology pulls more sensitive data from all of the photographs when compared to other methods in the literature. Our findings illustrate that the suggested strategy is a very valuable computational model. The goal is to evaluate this method in additional databases for future research, in addition to integrating new representations for application and investigation in dermoscopy images. As computer vision technology develops, machine learning is gaining popularity as a technique of automated medical picture recognition. Skin cancer screening techniques based on machine learning have been presented in the past research projects. However, this procedure produces high classification accuracy compared to other approaches. SDP and stacked RBM are employed in this work to categorize skin cancer photos. Different computational techniques are no longer required to extract color, texture, and edge information from skin cancer images. Clinicians will benefit from real-time skin cancer diagnosis if the suggested method can be implemented as a smartphone application.

## Figures and Tables

**Table 1:** The hyper-parameters in RBM.

The learning rate	0.1
The momentum	0.7
Highest count of epochs in training	300
Batch size	25
The Delay of gap stop	3
The Delay of momentum	0.7

**Table 2:** PH2 dataset confusion matrix.

Type	Total Amount of Test Images	Atypical Nevus (%)	Common Nevus (%)	Melanoma (%)	Total Positive Rate (TPR) (%)
**Atypical Nevus**	40	99	0	1	99
**Common Nevus**	40	0	100	0	100
**Melanoma**	20	4	0	96	96

**Table 3:** Classification evaluation performance for the PH2.

Descriptors	Classifier	SP	SE	PP	F-Score	HM	ACC ± SD
LBP	K-NN	97.0	92.4	80.1	88.4	85.4	89.3 ± 1.23
	Bayes	88.2	85.2	83.4	82.1	88.2	84.8 ± 0.25
	LS-SVM	92.7	92.5	88.3	92.5	92.1	92.8 ± 1.26
	ELM	89.2	82.3	93.2	82.1	79.0	91.9 ± 3.45
	MLP	91.9	78.2	88.3	82.1	78.1	78.2 ± 4.53
	Stacked RBM	99.2	99.4	96.3	97.4	96.6	81.1 ± 4.27
CLDP	K-NN	98.2	96.4	98.1	98.3	98.4	98.33 ± 0.44
	Bayes	90.1	87.6	89.4	87.8	88.2	87.2 ± 2.34
	LS-SVM(RBF)	94.7	92.9	90.3	94.5	94.1	94.8 ± 0.77
	ELM(RBF)	91.2	82.3	93.2	82.1	79.0	91.9 ± 0.83
	MLP	90.9	78.2	88.3	82.1	78.1	78.2 ± 2.34
	Stacked RBM	98.5	97.8	98.6	98.6	98.4	98.8 ± 4.03
Proposed SDP	K-NN	97.2	95.4	97.1	97.3	98.4	98.3 ± 2.24
	Bayes	91.2	87.2	89.1	86.5	87.6	87.1 ± 4.23
	LS-SVM(RBF)	94.7	89.9	89.3	93.5	93.1	93.8 ± 0.23
	ELM(RBF)	91.2	81.3	93.2	82.1	79.0	91.9 ± 4.03
	MLP	90.9	77.2	89.3	82.1	78.1	78.2 ± 5.23
	Stacked RBM	99.6	98.8	99.6	99.6	99.4	99.8 ± 1.20

**Table 4:** ISIC 2016 dataset confusion matrix.

Type	Total Amount of Test Images	Benign (%)	Melanoma (%)	TPR (%)
Benign type	250	100	0	100
Melanoma type	50	6	94	94

**Table 5:** Classification outcomes for the pictures of ISIC 2016.

Descriptor	Classifier	SP	SE	PP	F1-Score	HM	ACC ± SD
LBP	K-NN	83.0	82.4	81.1	76.3	71.3	71.3 ± 1.42
	Bayes	71.2	78.2	76.4	72.1	72.2	76.3 ± 0.44
	LS-SVM(RBF)	72.7	80.3	82.3	84.8	83.1	86.8 ± 1.45
	ELM(RBF)	71.2	82.1	84.2	82.5	68.0	68.5 ± 2.54
	MLP	71.9	79.2	89.3	82.1	87.1	79.2 ± 1.98
	Stacked RBM	**90.2**	**90.4**	**86.3**	**86.4**	**84.6**	**81.1** ± 7.14
CLDP	K-NN	88.2	86.4	90.1	98.3	88.4	90.3 ± 1.45
	Bayes	80.0	80.2	89.0	87.9	88.3	87.0 ± 5.44
	LS-SVM(RBF)	74.7	82.5	90.3	90.5	91.1	91.8 ± 2.57
	ELM(RBF)	81.2	72.3	90.2	82.1	79.0	91.9 ± 4.54
	MLP	80.9	78.2	88.3	82.1	78.1	78.2 ± 12.32
	Stacked RBM	88.5	92.1	93.3	92.6	93.0	95.5 ± 2.54
Proposed SDP	K-NN	80.2	85.4	91.1	99.3	89.4	92.3 ± 2.67
	Bayes	81.0	81.2	88.0	88.9	87.3	88.0 ± 5.54
	LS-SVM (RBF)	72.7	83.5	91.3	92.5	90.1	92.8 ± 2.22
	ELM (RBF)	83.2	71.3	91.2	83.1	77.0	90.9 ± 0.52
	MLP	81.9	79.2	89.3	81.1	76.1	79.2 ± 0.54
	Stacked RBM	**92.5**	**95.7**	**95.3**	**95.6**	**95.2**	**96.5** ± 2.54

**Table 6:** ISIC 2017 dataset confusion matrix.

Type	Total Amount of Test Images	Benign (%)	Melanoma (%)	TPR (%)
**Benign type**	308	96	4	96
**Melanoma type**	58	3	97	97

**Table 7:** Classification outcomes of pictures from ISIC 2017.

Descriptors	Classifier	SP	SE	PP	F-Score	HM	ACC ± SD
LBP	K-NN	71.0	62.4	65.1	66.3	62.3	60.3 ± 6.29
	Bayes	61.2	68.2	66.4	62.1	63.2	65.3 ± 4.22
	LS-SVM(RBF)	61.7	63.3	61.3	64.8	64.1	64.8 ± 2.29
	ELM(RBF)	62.2	64.0	64.2	62.3	59.0	51.5 ± 4.27
	MLP	64.5	69.2	67.3	62.1	68.0	68.2 ± 0.29
	Stacked RBM	79.2	79.4	69.3	69.3	75.6	80.1 ± 2.34
CLDP	K-NN	65.2	66.4	70.1	88.3	78.4	70.3 ± 5.45
	Bayes	60.0	60.2	79.0	77.9	78.3	77.0 ± 2.29
	LS-SVM (RBF)	61.7	62.5	70.3	80.5	81.1	71.8 ± 0.88
	ELM (RBF)	61.2	72.3	60.2	72.1	69.0	71.9 ± 6.23
	MLP	70.9	72.2	68.3	72.1	68.1	68.2 ± 0.29
	Stacked RBM	**96.5**	**98.1**	**80.3**	**82.7**	**83.2**	**94.5** ± 1.28
Proposed SDP	K-NN	66.2	77.4	72.1	88.3	78.4	70.3 ± 5.29
	Bayes	61.0	72.2	79.0	77.9	78.3	77.0 ± 4.29
	LS-SVM (RBF)	62.7	74.5	72.3	80.5	81.1	71.8 ± 6.23
	ELM (RBF)	63.2	73.3	62.2	72.1	69.0	71.9 ± 6.28
	MLP	72.9	72.2	68.3	72.1	68.1	68.2 ± 4.54
	Stacked RBM	**98.5**	**99.9**	**82.3**	**84.7**	**85.2**	**95.5** ± 2.12

**Table 8:** DermIS dataset confusion matrix.

Type	Total Amount of Test Images	Malignant Melanoma (%)	Seborrheic Keratosis (%)	Lupus Erythematosus (%)	TPR (%)
**Malignant Melanoma (%)**	18	97	2	1	97
**Seborrheic keratosis (%)**	48	0	99	1	99
**Lupus Erythematosus (%)**	24	3	0	97	97

**Table 9:** Classification outcomes of pictures from DermIS.

Descriptors	Classifier	SP	SE	PP	F-Score	HA	ACC ± SD
LBP	K-NN	75.6	61.4	6.1	66.3	62.3	60.3 ± 0.22
	Bayes	62.4	67.2	65.4	62.1	63.2	65.3 ± 2.22
	LS-SVM(RBF)	65.8	62.3	60.3	63.8	63.1	65.8 ± 4.22
	ELM(RBF)	64.2	62.0	63.2	76.3	65.0	61.5 ± 2.67
	MLP	65.5	68.2	68.3	62.1	68.0	68.2 ± 8.75
	Stacked RBM	69.9	79.4	67.3	68.3	77.6	83.1 ± 9.52
CLDP	K-NN	68.2	66.4	70.1	88.3	88.4	80.3 ± 0.29
	Bayes	56.0	90.2	89.0	67.9	88.3	87.0 ± 9.29
	LS-SVM(RBF)	82.7	92.5	90.1	90.1	81.1	81.8 ± 2.29
	ELM(RBF)	89.2	72.3	90.2	79.1	89.0	71.9 ± 0.44
	MLP	90.9	92.2	88.3	92.1	88.1	68.2 ± 0.56
	Stacked RBM	**95.9**	**99.9**	**90.3**	**92.7**	**83.2**	**96.6** + **3.44**
Proposed SDP	K-NN	68.2	66.4	70.1	88.3	88.4	80.3 ± 6.29
	Bayes	55.0	95.2	89.0	68.9	89.3	87.0 ± 5.44
	LS-SVM(RBF)	85.7	98.5	97.1	92.1	89.1	81.8 ± 5.67
	ELM(RBF)	89.2	72.3	92.2	79.1	89.0	72.9 ± 0.29
	MLP	91.9	93.2	89.3	93.1	89.1	68.2 ± 4.54
	Stacked RBM	**96.9**	**99.9**	**92.3**	**94.7**	**84.2**	**97.6** ± 3.67

**Table 10:** Dermnet dataset confusion matrix.

**Type**	**Total Amount of Test Images**	**Melanoma and Melanocytic Nevi (%)**	**Seborrhoeic Keratosis and Other Type of Benign Tumors (%)**	**Common Warts, Molluscum Contagiosum** **and Other (%)**	**TPR (%)**
Melanoma and Melanocytic Nevi (%)	635	89	10	1	89
Seborrhoeic Keratosis and other type of Benign Tumors (%)	2397	10	87	3	87
Common Warts, Molluscum contagiosum and other (%)	1549	9.5	1.5	89	89

**Table 11:** Classification outcomes of Dermnet.

Descriptors	Classifier	SP	SE	PP	F-score	HM	ACC ± SD
LBP	K-NN	72.3	61.4	69.1	66.3	62.3	60.3 ± 5.34
	Bayes	63.9	60.2	64.4	62.1	62.2	65.3 ± 3.87
	LS-SVM (RBF)	64.8	62.3	63.3	64.5	64.1	62.8 ± 0.29
	ELM (RBF)	64.2	67.0	61.4	76.3	69.0	62.5 ± 0.32
	MLP	65.5	62.2	64.3	62.1	68.0	65.2 ± 0.67
	Stacked RBM	76.9	78.4	75.2	76.3	75.6	80.1 ± 4.53
CLDP	K-NN	67.2	66.4	70.1	78.3	78.4	80.3 ± 6.34
	Bayes	72.0	70.2	78.0	67.9	78.3	77.0 ± 3.45
	LS-SVM(RBF)	72.7	81.5	70.1	71.1	81.1	71.8 ± 6.78
	ELM (RBF)	73.2	62.3	70.2	69.1	79.0	71.9 ± 5.89
	MLP	70.9	72.2	78.3	72.1	78.1	68.2 ± 0.54
	Stacked RBM	**98.9**	**87.8**	**86.1**	**88.5**	**85.2**	**87.0** ± 6.29
Proposed SDP	K-NN	68.2	66.4	70.1	78.3	78.4	80.3 ± 3.22
	Bayes	73.0	70.2	79.0	67.9	78.3	77.0 ± 2.23
	LS-SVM (RBF)	71.7	80.5	70.1	70.1	81.1	71.8 ± 5.67
	ELM (RBF)	71.2	62.3	70.2	69.1	79.0	71.9 ± 5.66
	MLP	71.9	72.2	78.3	72.1	78.1	68.2 ± 0.23
	Stacked RBM	**99.9**	**88.3**	**86.3**	**88.7**	**85.2**	**87.9** ± 2.33

## Data Availability

The following datasets are publicly available: (i) BCN_20000 Dataset from Department of Dermatology, Hospital Clínic de Barcelona; (ii) HAM10000 Dataset from ViDIR Group, Department of Dermatology, Medical University of Vienna; and (iii) MSK Dataset from Anonymous (accessed on January 2023).

## Supplementary Materials

The following supporting information can be downloaded at: https://www.mdpi.com/article/10.3390/diagnostics13061104/s1, Table S1: ISIC 2019 dataset confusion matrix; Table S2: Comparison with other cutting-edge methods.
