# Supplementary material for: A Hybrid Stacked Restricted Boltzmann Machine with Sobel Directional Patterns for Melanoma Prediction in Colored Skin Images
Source: Diagnostics (Basel). 2023 Mar 14;13(6):1104. doi: 10.3390/diagnostics13061104 (PMC10047753; doi:10.3390/diagnostics13061104)
Supplement: Supplementary file 1 [file diagnostics-13-01104-s001.zip › diagnostics-2220485-supplementary.pdf]

Supplementary Table S1. ISIC 2019 dataset confusion matrix.

Type	Total Amount of test images	Basal cell carcinoma (%)	Bowen disease (%)	Squamous cell carci-noma (%)	TPR (%)
Basal cell carcinoma	200	91	3.2	5.8	91
Bowen disease	200	7	83	10	83
Squamous cell carcinoma	200	20	18	62	62

Supplementary Table S2. Comparison with other cutting-edge methods.

	Approaches	SP	SE	AUC	ACC
**PH2**	Three dimensions [60]	97	96	-	95.75
	Multi-scaling [61]	93.13	87.5	-	92
	Texture-based feature extraction [62]	84	97	90	96
	CLDP and additional features [8]	97.8	98.5	98.3	98.8
	**Proposed method**	**99.6**	**98.8**	**99.3**	**99.8**
**ISIC 2016**	Skicit learn package [14]	92	50	67	91.6
	CNN using spatial domain [17]	94	50	80.4	85
	CNN [31]	79.7	78.6		81.33
	CNN [32]	-	-	-	83.9
	CNN based on regions [36]	-	94	98	94.4
	CLDP and additional features [9]	92.1	94.7	98.6	95.5
	**Proposed method**	**92.5**	**95.7**	**98.6**	**96.5**
**ISIC 2017**	Segmentation [16]	90.1	71.8	92.6	-
	Deep Neural Network [33]	-	-	95.8	-
	Deep learning (DL) approach [34]	93.3	50.4	82.3	85.2
	Structural co-occurrence matrix [34]	89.9	92.15	89	89.93
	DL [35]	**-**	-	-	93.29
	CNN based on regions [36]	**-**	94.2	98	93.4
	CLDP and additional features [9]	96.5	98.1	98.6	94.5
	**Proposed method**	**98.5**	**99.9**	**98.6**	**95.5**
**Dermnet**	DNN [37]	97.8	82.5	-	-
	CLDP and additional features [9]	**98.9**	**87.9**		**87.0**
	**Proposed method**	**99.9**	**88.3**		**87.9**
**DermIS**	Bat-optimization algorithm [38]	91.77	99.27	-	96.28
	CLDP and additional features [9]	95.9	99.9		96.6
	**Proposed method**	**96.9**	**99.9**		**97.6**
